# A statistical inference framework for FSNBLR: Modeling underdeveloped regional status in Eastern Indonesia^☆^

**DOI:** 10.1016/j.mex.2025.103746

**Published:** 2025-12-01

**Authors:** Muhammad Zulfadhli, I Nyoman Budiantara, Vita Ratnasari, Afiqah Saffa Suriaslan, Risdiana Chandra Dhewy

**Affiliations:** Departement of Statistics, Institut Teknologi Sepuluh Nopember, Kampus ITS-Sukolilo, Surabaya 60111, Indonesia

**Keywords:** Bernoulli distribution, Binary logistic regression (BLR), Categorical data, Fourier series nonparametric binary logistic regression (FSNBLR), Hypothesis test, Likelihood ratio test (LRT), Underdeveloped regions

## Abstract

Persistent regional disparities in Indonesia, particularly in Eastern provinces, necessitate advanced modeling to understand underdevelopment determinants. This study enhances the Fourier Series Nonparametric Binary Logistic Regression (FSNBLR) model by introducing a statistical inference framework comprising simultaneous and partial hypothesis testing using the Likelihood Ratio Test (LRT). Applying the model to data from 232 regencies in Eastern Indonesia (2021) identifies infrastructure quality and local fiscal capacity as significant predictors of underdevelopment. Compared with the conventional Binary Logistic Regression (BLR), the FSNBLR with significant parameters demonstrates superior classification accuracy and lower AIC values, effectively capturing nonlinear relationships among predictors. The proposed framework strengthens the inferential foundation of FSNBLR and broadens its applicability to complex binary response analyses in socioeconomic studies. The highlights of this study are:

Developed inferential hypothesis testing for the FSNBLR model.

Implemented LRT for simultaneous and partial inference.

The FSNBLR model outperforms BLR model in capturing nonlinearities.

## Graphical abstract

**Figure:**
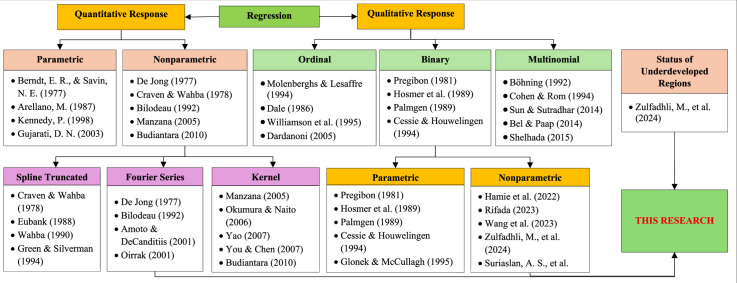

## Specifications table

**Table:** 

Subject area	Mathematics and Statistics
More specific subject area	Statistics; Nonparametric Regression; Categorical Data
Name of your method	Fourier Series Nonparametric Binary Logistic Regression (FSNBLR)
Name and reference of original method	Fourier series function developed by Bilodeau (1992), M. Bilodeau, Fourier smoother and additive models, The Canadian Journal of Statistics 20 (1992) 257–269. https://doi.org/10.2307/3315313. Maximum likelihood estimation in the book of Hosmer, D. W., & Lemeshow, S. (2000), Applied Logistic Regression, 2nd Edition. United States of America, Canada. https://books.google.co.id/books?id=Po0RLQ7USIMC&lpg=PP1&pg=PP1#v=onepage&q&f=false
Resource availability	Return on asset and its predictor variables data in 2021 of 232 districities/municipalities in East Indonesia could be accessed at the Indonesia publication data website (https://www.bps.go.id/id).

## Background

Regional inequality remains a persistent challenge in many developing countries, including Indonesia. In particular, several districts across Eastern Indonesia continue to be classified as underdeveloped regions, characterized by deficiencies in infrastructure, education, economic activity, and access to basic services. This persistent disparity reflects Indonesia’s broader structural inequality, rooted in historical concentration of economic activities, fiscal resources, and infrastructure investment in Western Indonesia—particularly Java and Sumatra—while Eastern provinces such as Papua, Maluku, and East Nusa Tenggara have experienced slower structural transformation and limited access to development opportunities. Despite numerous government initiatives, including the Masterplan for Acceleration and Expansion of Indonesia’s Economic Development (MP3EI) and the Village Fund Program, these regions continue to lag behind due to geographic isolation, institutional capacity constraints, and uneven policy implementation.

Consequently, Eastern Indonesia provides a distinctive and policy-relevant context for analyzing the determinants of regional underdevelopment, where socioeconomic, geographic, and institutional factors intersect most sharply. Understanding the underlying determinants of underdevelopment is critical for informing targeted and effective policy interventions. However, the classification of regions as underdeveloped is often influenced by a complex interplay of multiple socioeconomic, geographic, and institutional factors. This complexity presents a significant modeling challenge, especially when conventional statistical models are insufficient to capture nonlinear or nonstandard relationships among variables.

Recent methodological advancements have led to the adoption of more flexible modeling approaches, including nonparametric regression frameworks. Among them, the Fourier Series Nonparametric Binary Logistic Regression (FSNBLR) model has emerged as a promising technique for modeling binary outcomes without imposing restrictive assumptions on the functional form of the relationship between predictors and the response variable [1]. FSNBLR builds upon the foundation of Nonparametric Binary Logistic Regression (NBLR), which was introduced to capture complex associations when the regression function is unknown or difficult to specify a prior [2]. Unlike traditional Binary Logistic Regression (BLR), which relies on the assumption of a linear link between predictors and the logit-transformed response, NBLR and its variants utilize data-driven smoothing techniques to estimate the regression function, thus offering greater modeling flexibility.

Generalized Additive Models (GAMs), provide a flexible framework by modeling the predictors' effects through smooth functions. However, GAMs often rely on spline-based smoothers and do not exploit the analytical advantages of orthogonal basis functions like Fourier series. Additionally, GAMs usually require careful selection of smoothing parameters and may not be optimized for periodic structures in the data [3]. The proposed FSNBLR addresses these limitations by introducing a Fourier basis within a nonparametric logistic regression setting, allowing for compact, interpretable representations of nonlinear patterns—particularly suitable for modeling periodic or oscillatory behavior.

Numerous smoothing techniques have been developed to operationalize nonparametric regression models, including the Spline estimator [4], Fourier Series estimator [5], Wavelet estimator [6], Kernel estimator [7], Local Polynomial Regression [8], and Multivariate Adaptive Regression Splines (MARS) [9]. Each method is suited to particular data structures and research contexts. For instance, splines are effective for data exhibiting localized variation based on predefined knots [4], while local polynomial regression has been employed to reduce bias and asymptotic variance in multivariate contexts [10]. Wavelet-based methods have demonstrated robustness in handling noisy data, particularly when the error component follows a Gaussian distribution [11,12].

Beyond the statistical literature, studies in development economics and spatial inequality have highlighted how disparities in fiscal capacity, human capital, and infrastructure investment contribute to persistent regional underdevelopment [[13], [14], [15]]. Empirical analyses such as those by [13] and [14] emphasize that Indonesia’s regional inequality is shaped by both structural and institutional dimensions, underscoring the need for data-driven modeling tools that can capture complex, nonlinear interdependencies. Integrating flexible statistical approaches like FSNBLR with regional policy research thus provides a stronger analytical foundation for identifying which socioeconomic variables most effectively inform spatially targeted development interventions.

Among these approaches, the Fourier Series estimator is particularly noteworthy for its ability to model periodic or repeating patterns in data [4]. It has demonstrated a favorable balance between model accuracy and computational efficiency in additive nonparametric regression frameworks [16]. Furthermore, it has shown strong performance in both univariate and multivariate settings [5,[16], [17], [18]]. Since its initial development in [5] and later expansion in [11], Fourier-based methods have been extended to semiparametric regression models [19], adapted for bivariate responses [20], and incorporated into various mixture-type modeling frameworks in both nonparametric [[21], [22], [23]] and semiparametric forms [24].

Despite its strengths, most applications of Fourier-based estimators have focused on continuous response variables [[25], [26], [27], [28]]. In practice, however, many empirical problems—such as classifying regions as underdeveloped or not—require models suited for binary or categorical outcomes. In response, researchers have explored nonparametric regression approaches for categorical data, including Local Likelihood Logit Estimation [29], decision tree-based methods [30], and B-spline-based techniques [31]. More recently, the FSNBLR model has been proposed as a nonparametric extension specifically tailored for binary outcomes [1].

However, existing applications of FSNBLR have primarily emphasized its descriptive capabilities and estimation flexibility, without incorporating the necessary inferential framework for hypothesis testing and formal assessment of predictor significance. This limitation hinders its utility in applied settings, where determining which predictors have statistically significant effects on binary outcomes is essential for evidence-based decision-making. Addressing this gap, the present study proposes a comprehensive inferential framework for the FSNBLR model, extending its functionality beyond estimation to support statistical inference. Specifically, the study introduces two hypothesis testing procedures: (i) simultaneous hypothesis testing via the Likelihood Ratio Test (LRT), which yields a test statistic asymptotically following a Chi-square distribution; and (ii) partial (individual) hypothesis testing via the Z-test, which evaluates the significance of individual predictors within the nonparametric structure.

In addition, this study formulates explicit research hypotheses grounded in socioeconomic development theory, positing that regions with higher fiscal capacity, better infrastructure, and greater human capital are less likely to be classified as underdeveloped, thereby linking the FSNBLR modeling framework with the theoretical underpinnings of regional inequality. To illustrate the practical utility of the proposed methodology, the FSNBLR model and its inferential extensions are applied to an empirical case study examining the determinants of regional underdevelopment in Eastern Indonesia. The analysis employs secondary data comprising one binary outcome variable (indicating underdeveloped status) and multiple predictor variables reflecting regional development indicators.

This study contributes to the literature in two key dimensions. Methodologically, it advances the FSNBLR framework by integrating formal statistical inference tools, enabling researchers to conduct hypothesis testing within a flexible, nonparametric binary regression context. Practically, it offers empirical insights into the drivers of regional underdevelopment, thereby providing a more rigorous and data-informed basis for regional policy planning and targeted development strategies in Indonesia. FSNBLR models such as this can provide classification results that correspond to data patterns and the significance of model parameters in identifying variables that can be prioritized by policymakers.

## Method details

The development of the hypothesis testing procedure for the FSNBLR estimator involves several essential steps. First, the probability distribution of the binary response variable will be formulated. Based on this distribution, the FSNBLR model will then be constructed by incorporating the Fourier Series into the logistic regression framework. Subsequently, statistical inference will be established through the formulation of appropriate hypothesis testing procedures. In this context, simultaneous hypothesis testing will be conducted using the LRT, which evaluates the joint significance of the model parameters. Meanwhile, partial hypothesis testing will be carried out using the Wald test, which assesses the individual significance of each predictor variable. Conceptually, the use of the Fourier basis within the FSNBLR framework allows complex and nonlinear relationships to be expressed as a combination of smooth, periodic components. This is particularly suitable for socioeconomic phenomena that often exhibit cyclical or recurrent patterns—such as fiscal cycles, migration flows, or seasonal economic activities—where relationships between predictors and outcomes are rarely linear. By decomposing these relationships into interpretable harmonic terms, the FSNBLR model can capture nuanced variations in the data while remaining flexible and transparent, thereby making the methodology more accessible to researchers and policymakers beyond the field of statistics.

### Probability distribution

Given$\;x_{1i},\;x_{2i}\ldots x_{pi}\;;i=1,\;2...\;n$, representing p predictor variables, the response variableYwas assumed to follow a Bernoulli distribution [1] with a probability distribution defined as:

$$Y\;∼\;B\left(1,\pi \left(x\right)\right),\;\pi \left(x\right)=\pi \left(x_{1},\;x_{2}\ldots \;x_{p}\right)$$

where the success probability was given by:

$$P\left(Y_{i}\;=\;1\right)=\pi \left(x_{i}\right)$$

and the failure probability by:

$$P\left(Y_{i}\;=\;0\right)=1-\pi \left(x_{i}\right)$$

The probability function$\;\pi (x_{i})\;$was defined in the Bernoulli probability mass function as follows:

(1) $$P\left(Y_{i}\;=\;y_{i}\right)=\pi {\left(x_{i}\right)}^{y_{i}}{\left(1-\pi \left(x_{i}\right)\right)}^{1-y_{i}}={\left(\frac{\pi \left(x_{i}\right)}{1-\pi \left(x_{i}\right)}\right)}^{y_{i}}\left(1-\pi \left(x_{i}\right)\right)$$

### FSNBLR estimator

#### FSNBLR model

The FSNBLR model is obtained [31] as follows:

(2) $$\pi \left(x_{i}\right)=\;\frac{\exp\left(\sum_{j=1}^{p}\left(b_{j}x_{ji}+\frac{1}{2}a_{0j}+\sum_{k=1}^{K}a_{kj}\cos kx_{ji}\right)\right)}{1+\exp\left(\sum_{j=1}^{p}\left(b_{j}x_{ji}+\frac{1}{2}a_{0j}+\sum_{k=1}^{K}a_{kj}\cos kx_{ji}\right)\right)}\;;\;i=1,2...n$$

In the model (2),$\;b_{j},a_{0j}\;\text{and}\;a_{kj}\;$, wherej=1,2…p, wherek=1,2…K, are the Fourier Series model parameters. Estimator of the model can be obtained using the Maximum Likelihood Estimation (MLE) method.

#### Likelihood functionl(θ)

The likelihood functionl(θ)is given, where

$$\theta =\left(\begin{array}{ccc} b_{1} & a_{01} & \begin{array}{ccc} a_{11} & \ldots & \begin{array}{ccc} a_{K1} & \vdots & \begin{array}{ccc} \cdots & \vdots & \begin{array}{ccc} b_{p} & a_{0p} & \begin{array}{ccc} a_{1p} & \ldots & a_{Kp} \end{array} \end{array} \end{array} \end{array} \end{array} \end{array}\right)$$

The likelihood function which is the multiplication of joint probabilities of random variables (1) is formulated as:

(3) $$l\left(\theta \right)=\;\prod_{i=1}^{n}P\left(Y_{i}=\;y_{i}\right)=\pi {\left(x_{i}\right)}^{\sum_{i=1}^{n}y_{i}}{\left(1-\pi \left(x_{i}\right)\right)}^{n-\sum_{i=1}^{n}y_{i}}\;$$

In practice, the parameter estimation in logistic regression is conducted by maximizing the log-likelihood function, which is obtained by taking the natural logarithm of Eq. (3). This transformation simplifies the optimization process, and the log-likelihood function is written aslnl(θ).

#### Log-likelihood functionL(θ)

The likelihood functionL(θ)is expressed as follow:

(4) $$\begin{array}{c} L\left(\theta \right)=\ln l\left(\theta \right)=\sum_{i=1}^{n}y_{i}\ln\left[\pi {\left(x_{i}\right)}^{\;}\right]+\sum_{i=1}^{n}\left(1-y_{i}\right)\ln\left[1-\pi {\left(x_{i}\right)}^{\;}\right]\;\\ =\;\sum_{i=1}^{n}\left\{y_{i}\left(f\left(x_{1i},\;\ldots \;,\;x_{pi}\right)\right)-\ln\left[1+\exp(f\left(x_{1i}\ldots \;x_{pi}\right))\right]\right\}\; \end{array}$$

The estimator$\;\hat{\theta }\;$derived by taking the partial derivatives of the log-likelihood function (4) with respect to each parameter$\;b_{j},a_{0j},a_{kj}$, and equating these derivatives to zero.

#### Newton-Raphson iteration

The derivative of the log-likelihood functionL(θ)in Eq. (4) with respect to the parameters$\;b_{j},a_{0j},a_{kj}\;$produces a complex system of equations that has no closed-form solution. This means that we cannot directly calculate the parameter values explicitly. Therefore, we need to use numerical methods to find the optimal parameter values. One method used is the Newton–Raphson method, which works iteratively to estimate the best values of the parameter vectorθ. The iterative updating equation is given by:

(5) $$\theta^{\left(t+1\right)}=\;\theta^{\left(t\right)}-\;{\left(H{\left(\theta \right)}^{\left(t\right)}\right)}^{-1}g{\left(\theta \right)}^{\left(t\right)}\;$$

where$\;\theta^{\left(t\right)}\;$in Eq. (5) is the parameter estimate at the *t*-th iteration fort=1,2…until convergence. The vector$\;\theta^{\left(t\right)}\;$is defined as:

$$\theta^{\left(t\right)}=\left(\begin{array}{ccc} {b_{1}}^{\left(t\right)} & {a_{01}}^{\left(t\right)} & \begin{array}{ccc} {a_{11}}^{\left(t\right)} & \ldots & \begin{array}{ccc} {a_{K1}}^{\left(t\right)} & \vdots & \begin{array}{ccc} \cdots & \vdots & \begin{array}{ccc} {b_{p}}^{\left(t\right)} & {a_{0p}}^{\left(t\right)} & \begin{array}{ccc} {a_{1p}}^{\left(t\right)} & \ldots & {a_{Kp}}^{\left(t\right)} \end{array} \end{array} \end{array} \end{array} \end{array} \end{array}\right)$$

The gradient vectorg(θ)contains the first-order partial derivatives of the log-likelihood function and is defined (6) as:

(6) $$g\left(\theta \right)=\;{\left(\frac{\partial L\left(\theta \right)}{\partial b_{1}},\frac{\partial L\left(\theta \right)}{\partial a_{01}},\frac{\partial L\left(\theta \right)}{\partial a_{11}}\cdots \frac{\partial L\left(\theta \right)}{\partial a_{K1}}\cdots \frac{\partial L\left(\theta \right)}{\partial b_{p}},\frac{\partial L\left(\theta \right)}{\partial a_{0p}},\frac{\partial L\left(\theta \right)}{\partial a_{1p}}\cdots \frac{\partial L\left(\theta \right)}{\partial a_{Kp}}\right)}^{T}\;$$

The Hessian matrixH(θ)is composed of the second-order partial derivatives of the log-likelihood function and is organized (7) as follows:

(7) $$H\left(\theta \right)\;=\left[\begin{array}{cc} \begin{array}{cc} \frac{\partial^{2}L\left(\theta \right)}{\partial {b_{1}}^{2}} & \frac{\partial^{2}L\left(\theta \right)}{\partial b_{1}\partial a_{01}}\\ \frac{\partial^{2}L\left(\theta \right)}{\partial a_{01}\partial b_{1}} & \frac{\partial^{2}L\left(\theta \right)}{\partial {a_{01}}^{2}} \end{array} & \begin{array}{cc} \cdots & \frac{\partial^{2}L\left(\theta \right)}{\partial b_{1}\partial a_{Kp}}\\ \cdots & \frac{\partial^{2}L\left(\theta \right)}{\partial a_{01}\partial a_{Kp}} \end{array}\\ \begin{array}{cc} \vdots & \vdots \\ \frac{\partial^{2}L\left(\theta \right)}{\partial a_{Kp}\partial b_{1}} & \frac{\partial^{2}L\left(\theta \right)}{\partial a_{Kp}\partial a_{01}} \end{array} & \begin{array}{cc} \ddots & \vdots \\ \cdots & \frac{\partial^{2}L\left(\theta \right)}{\partial {a_{Kp}}^{2}} \end{array} \end{array}\right]\;$$

Here,$\;b_{j},a_{0j}\;\text{and}\;a_{kj}\;,\;j,u=1,2\ldots \;p,\;j\neq u,k=1,2\ldots K\;$are the model parameters associated with the Fourier Series expansion within the nonparametric regression framework.

#### Estimator$\;\hat{\theta }$

From the Newton-Raphson iteration equation, the estimator$\;\hat{\theta }\;$is obtained when the convergence criterion is met:

(8) $$\left|\theta^{\left(t+1\right)}-\theta^{\left(t\right)}\right|<\varepsilon ,\;\varepsilon =0,000001\;$$

Once this condition is satisfied (8), the final estimator vector$\;\hat{\theta }\;$is expressed as:

$$\hat{\theta }=\left(\begin{array}{ccc} {\hat{b}}_{1} & {\hat{a_{0}}}_{1} & \begin{array}{ccc} {\hat{a_{1}}}_{1} & \ldots & \begin{array}{ccc} {\hat{a_{K}}}_{1} & \vdots & \begin{array}{ccc} \cdots & \vdots & \begin{array}{ccc} {\hat{b}}_{p} & {\hat{a_{0}}}_{p} & \begin{array}{ccc} {\hat{a_{1}}}_{p} & \ldots & {\hat{a_{K}}}_{p} \end{array} \end{array} \end{array} \end{array} \end{array} \end{array}\right)$$

Based on the resulting estimator$\;\hat{\theta }$, the FSNBLR model (2) can be written as:

(9) $$\hat{\pi }\left(x_{i}\right)=\;\frac{\text{exp}\left({\hat{b}}_{1}x_{1i}+\frac{1}{2}{\hat{a}}_{01}+{\hat{a}}_{11}\cos x_{1i}+\ldots +{\hat{a}}_{K1}\cos Kx_{1i}+\ldots +{\hat{b}}_{p}x_{pi}+\frac{1}{2}{\hat{a}}_{0p}+{\hat{a}}_{1p}\cos x_{pi}+\ldots +{\hat{a}}_{Kp}\cos Kx_{pi}\right)}{1+\exp\left({\hat{b}}_{1}x_{1i}+\frac{1}{2}{\hat{a}}_{01}+{\hat{a}}_{11}\cos x_{1i}+\ldots +{\hat{a}}_{K1}\cos Kx_{1i}+\ldots +{\hat{b}}_{p}x_{pi}+\frac{1}{2}{\hat{a}}_{0p}+{\hat{a}}_{1p}\cos x_{pi}+\ldots +{\hat{a}}_{Kp}\cos Kx_{pi}\right)}\;$$

Here,$\;{\hat{b}}_{1},{\hat{a}}_{01}\;\text{and}\;{\hat{a}}_{k1}\;$are the estimated model parameters associated with the predictor variable$\;x_{1}$, while$\;{\hat{b}}_{p},{\hat{a}}_{0p}\;\text{and}\;{\hat{a}}_{kp}\;$correspond to those associated with the predictor variable$\;x_{p}$. Wherepdenotes the total number of predictor variables andKrepresents the number of oscillation parameters and.

### Hypothesis test for parameter model

The hypothesis testing for model parameters is divided into two types: simultaneous and partial. The simultaneous test is conducted using the Likelihood Ratio Test (LRT), while the partial test is conducted using the Wald test.

#### Simultaneous test

The simultaneous (overall) test evaluates the significance of the full set of model parametersβin the model. The hypothesis form, parameter space, and statistic test are given as follows.

Hypothesis:

$H_{0}:\;b_{1}=b_{2}=\cdots =b_{p}=a_{11}=a_{21}=\cdots =a_{K1}=\cdots =a_{Kp}=0$
$H_{1}:\text{at}\;\text{least}\;\text{one}\;b_{j}\neq 0\;\text{or}\;a_{kj}\neq 0\;,\;k=1,2,\ldots ,K\;,\;j=1,2,\ldots ,p$

Parameter Spaces:

Under$\;H_{0}:\omega =\left\{{a_{0}}^{*}\right\}$

Under population:$\;\Omega =\{{a_{0}}^{*},b_{1}\cdots b_{p},a_{11}\cdots a_{K1}\cdots a_{Kp}\}$

Theorem 1. LetΩbe the parameter space for the population, and letω⊆Ωbe the parameter space under the null hypothesis$\;H_{0}$. Then, the test statistic used to test this hypothesis, called the Likelihood Ratio Test (LRT), is defined as follows:

(10) $$G^{2}=-2\ln\Lambda =-2\ln\left(\frac{\max_{\omega }l\left(\omega \right)}{\max_{\omega }l\left(\Omega \right)}\right)=\;2\left[\ln l\left({\hat{\theta }}_{\Omega }\right)-\ln l\left(\theta_{\omega }\right)\right]-2\left[\ln l\left({\hat{\theta }}_{\omega }\right)-\ln l\left(\theta_{\omega }\right)\right]$$

Proof:

Lemma 1.1. The log-likelihood function$\;\ln l(\theta_{\omega })\;$(10) in Theorem 1 is approximated using a second-order Taylor series expansion around the MLE under the full model$\;{\hat{\theta }}_{\Omega }$

(11) $$\ln l\left(\theta_{\omega }\right)\approx \ln l\left({\hat{\theta }}_{\Omega }\right)+g\left({\hat{\theta }}_{\Omega }\right)\left(\theta_{\omega }-{\hat{\theta }}_{\Omega }\right)-\frac{1}{2}{\left(\theta_{\omega }-{\hat{\theta }}_{\Omega }\right)}^{T}\left[I\left({\hat{\theta }}_{\Omega }\right)\right]\left(\theta_{\omega }-{\hat{\theta }}_{\Omega }\right)$$

With$\;I({\hat{\theta }}_{\Omega })\;$is the Fisher information matrix evaluated at$\;{\hat{\theta }}_{\Omega }$.

$$g\left({\hat{\theta }}_{\Omega }\right)=\frac{\partial \ln l\left(\theta_{\Omega }\right)}{\partial \theta_{\Omega }}{|}_{\theta_{\Omega }={\hat{\theta }}_{\Omega }}=0,\;I\left({\hat{\theta }}_{\Omega }\right)=-\frac{\partial^{2}\ln l\left(\theta_{\Omega }\right)}{\partial \theta_{\Omega }\partial {\theta_{\Omega }}^{T}}{|}_{\theta_{\Omega }={\hat{\theta }}_{\Omega }}$$

Corollary 1.1.1. Because in Eq. (11)$\;g({\hat{\theta }}_{\Omega })=0$, the formulation in Lemma 1.1 could be defined as follows:

(12) $$\begin{array}{c} \ln l\left(\theta_{\omega }\right)\approx \ln l\left({\hat{\theta }}_{\Omega }\right)-\frac{1}{2}{\left(\theta_{\omega }-{\hat{\theta }}_{\Omega }\right)}^{T}\left[I\left({\hat{\theta }}_{\Omega }\right)\right]\left(\theta_{\omega }-{\hat{\theta }}_{\Omega }\right)\\ 2\ln l\left({\hat{\theta }}_{\Omega }\right)-2\ln l\left(\theta_{\omega }\right)\approx \left(-{\left(\theta_{\omega }-{\hat{\theta }}_{\Omega }\right)}^{T}\right)\left[I\left({\hat{\theta }}_{\Omega }\right)\right]\left(-\left(\theta_{\omega }-{\hat{\theta }}_{\Omega }\right)\right)\\ 2\left[\ln l\left({\hat{\theta }}_{\Omega }\right)-\ln l\left(\theta_{\omega }\right)\right]\approx {\left({\hat{\theta }}_{\Omega }-\theta_{\omega }\right)}^{T}\left[I\left({\hat{\theta }}_{\Omega }\right)\right]\left({\hat{\theta }}_{\Omega }-\theta_{\omega }\right)\; \end{array}$$

The log-likelihood function$\;\ln l(\theta_{\omega })\;$expanding around$\;{\hat{\theta }}_{\omega }\;$gives:

$$\ln l\left(\theta_{\omega }\right)\approx \ln l\left({\hat{\theta }}_{\omega }\right)+g\left({\hat{\theta }}_{\Omega }\right)\left(\theta_{\omega }-{\hat{\theta }}_{\omega }\right)-\frac{1}{2}{\left(\theta_{\omega }-{\hat{\theta }}_{\omega }\right)}^{T}\left[I\left({\hat{\theta }}_{\Omega }\right)\right]\left(\theta_{\omega }-{\hat{\theta }}_{\omega }\right)$$

Corollary 1.1.2. Because$\;g({\hat{\theta }}_{\Omega })=0$, the formulation in Lemma 1.2 is given as follows:

(13) $$\begin{array}{c} \ln l\left(\theta_{\omega }\right)\approx \ln l\left({\hat{\theta }}_{\omega }\right)-\frac{1}{2}{\left(\theta_{\omega }-{\hat{\theta }}_{\omega }\right)}^{T}\left[I\left({\hat{\theta }}_{\Omega }\right)\right]\left(\theta_{\omega }-{\hat{\theta }}_{\omega }\right)\\ 2\ln l\left(\theta_{\omega }\right)\approx 2\ln l\left({\hat{\theta }}_{\omega }\right)-{\left(\theta_{\omega }-{\hat{\theta }}_{\omega }\right)}^{T}\left[I\left({\hat{\theta }}_{\Omega }\right)\right]\left(\theta_{\omega }-{\hat{\theta }}_{\omega }\right)\\ 2\left[\ln l\left({\hat{\theta }}_{\omega }\right)-\ln l\left(\theta_{\omega }\right)\right]\approx {\left({\hat{\theta }}_{\omega }-\theta_{\omega }\right)}^{T}\left[I\left({\hat{\theta }}_{\Omega }\right)\right]\left({\hat{\theta }}_{\omega }-\theta_{\omega }\right)\; \end{array}$$

Based on Lemma 1.1, combining (12) and (13), the LRT statistic in Theorem 1 becomes:

(14) $$\begin{array}{c} G^{2}=\;2\left[\ln l\left({\hat{\theta }}_{\Omega }\right)-\ln l\left(\theta_{\omega }\right)\right]-2\left[\ln l\left({\hat{\theta }}_{\omega }\right)-\ln l\left(\theta_{\omega }\right)\right]\\ G^{2}\approx \;{\left({\hat{\theta }}_{\Omega }-\theta_{\omega }\right)}^{T}\left[I\left({\hat{\theta }}_{\Omega }\right)\right]\left({\hat{\theta }}_{\Omega }-\theta_{\omega }\right)-{\left({\hat{\theta }}_{\omega }-\theta_{\omega }\right)}^{T}\left[I\left({\hat{\theta }}_{\Omega }\right)\right]\left({\hat{\theta }}_{\omega }-\theta_{\omega }\right) \end{array}$$

Suppose the parameters of the MLE estimator on the population are partitioned:

$${\hat{\theta }}_{\Omega }={\left[\begin{array}{cc} {\hat{\theta }}_{1} & {\hat{\theta }}_{2} \end{array}\right]}^{T}$$

where,

$${\hat{\theta }}_{1}={\left[\begin{array}{ccc} \begin{array}{ccc} {a_{0}}^{*} & b_{1} & \cdots \end{array} & \begin{array}{ccc} b_{p} & a_{11} & \cdots \end{array} & \begin{array}{ccc} a_{K1} & \cdots & a_{Kp} \end{array} \end{array}\right]}^{T}$$

$${\hat{\theta }}_{2}={\left[{a_{0}}^{*}\right]}^{T}$$

Suppose the parameters of the MLE estimator at$\;H_{0}\;$are partitioned:

$${\hat{\theta }}_{\omega }={\left[\begin{array}{cc} {\hat{\theta }}_{0} & {\hat{\theta }}_{0\omega } \end{array}\right]}^{T}$$

where,

$${\hat{\theta }}_{0}={\left[\begin{array}{ccc} 0 & 0 & \begin{array}{cc} \cdots & 0 \end{array} \end{array}\right]}^{T}=0_{p\left(K+1\right)\times \;1}$$

$${\hat{\theta }}_{0\omega }={\left[{a_{0}}^{**}\right]}^{T}$$

Lemma 1.2. Based on [32], the maximum likelihood estimator$\;{\hat{\theta }}_{\Omega }\;$satisfies the asymptotic distribution:

$$\left({\hat{\theta }}_{\Omega }-\theta_{\Omega }\right)\overset{d}{\to }N\left(0,{\left[I\left({\hat{\theta }}_{\Omega }\right)\right]}^{-1}\right)$$

and$\;{\hat{\theta }}_{\Omega }={\left[\begin{array}{cc} {\hat{\theta }}_{1} & {\hat{\theta }}_{2} \end{array}\right]}^{T}$, then obtained

$$\left(\begin{array}{c} {\hat{\theta }}_{1}-\theta_{1}\\ {\hat{\theta }}_{2}-\theta_{2} \end{array}\right)\overset{d}{\to }N\left(0,{\left[I\left({\hat{\theta }}_{\Omega }\right)\right]}^{-1}\right)$$

where,

$$I\left({\hat{\theta }}_{\Omega }\right)=\left[\begin{array}{cc} \left[I_{11}\right] & \left[I_{12}\right]\\ \left[I_{21}\right] & \left[I_{22}\right] \end{array}\right]$$

$${\left[I\left({\hat{\theta }}_{\Omega }\right)\right]}_{\left(\left(p\left(K+1\right)+1\right)\times \left(p\left(K+1\right)+1\right)\right)}=\left[\begin{array}{cc} {\left[I_{11}\right]}_{\left(\left(p\left(K+1\right)\right)\times \left(p\left(K+1\right)\right)\right)} & {\left[I_{12}\right]}_{\left(\left(p\left(K+1\right)\right)\times \;1\right)}\\ {\left[I_{21}\right]}_{\left(1\;\times \left(p\left(K+1\right)\right)\right)} & {\left[I_{22}\right]}_{1\;\times \;1} \end{array}\right]$$

and

$${\left[I\left({\hat{\theta }}_{\Omega }\right)\right]}^{-1}=\left[\begin{array}{cc} \left[I^{11}\right] & \left[I^{12}\right]\\ \left[I^{21}\right] & \left[I^{22}\right] \end{array}\right]$$

Corollary 1.2.1. According to [33], the matrix$\;{\left[I({\hat{\theta }}_{\Omega })\right]}^{-1}\;$can be written in the form of non singular matrices as follows:

$${\left[I\left({\hat{\theta }}_{\Omega }\right)\right]}^{-1}=\left[\begin{array}{cc} {\left[I_{11.2}\right]}^{-1} & -{\left[I_{11.2}\right]}^{-1}\left[I_{12}\right]{\left[I_{22}\right]}^{-1}\\ -{\left[I_{22}\right]}^{-1}\left[I_{21}\right]{\left[I_{11.2}\right]}^{-1} & {\left[I_{22.1}\right]}^{-1} \end{array}\right]$$

with$\;\left[I_{11.2}\right]=-\left[I_{11}\right]-\left[I_{12}\right]{\left[I_{22}\right]}^{-1}\left[I_{21}\right]\;$dan$\;{\left[I_{11}\right]}^{-1}=\left[I_{11.2}\right]$

The quadratic form$\;{\left({\hat{\theta }}_{\Omega }-\theta_{\omega }\right)}^{T}\left[I({\hat{\theta }}_{\Omega })\right]\left({\hat{\theta }}_{\Omega }-\theta_{\omega }\right)\;$in Eq. (14) can be decomposed into:

(15) $${\left({\hat{\theta }}_{\Omega }-\theta_{\omega }\right)}^{T}\left[I\left({\hat{\theta }}_{\Omega }\right)\right]\left({\hat{\theta }}_{\Omega }-\theta_{\omega }\right)={\left[\begin{array}{c} {\hat{\theta }}_{1}\\ {\hat{\theta }}_{2}-\theta_{0\omega } \end{array}\right]}^{T}\left[\begin{array}{cc} \left[I_{11}\right] & \left[I_{12}\right]\\ \left[I_{21}\right] & \left[I_{22}\right] \end{array}\right]\left[\begin{array}{c} {\hat{\theta }}_{1}\\ {\hat{\theta }}_{2}-\theta_{0\omega } \end{array}\right]$$

Corollary 1.2.2. Based on$\;{\hat{\theta }}_{\omega }={\left[\begin{array}{cc} {\hat{\theta }}_{0} & {\hat{\theta }}_{0\omega } \end{array}\right]}^{T}$, then obtained:

$$\left(\begin{array}{c} 0_{p\left(K+1\right)\times \;1}-0_{p\left(K+1\right)\times \;1}\\ {\hat{\theta }}_{0\omega }-\theta_{0\omega } \end{array}\right)\overset{d}{\to }N\left(0,{\left[I\left({\hat{\theta }}_{\Omega }\right)\right]}^{-1}\right)$$

The quadratic form$\;{\left({\hat{\theta }}_{\omega }-\theta_{\omega }\right)}^{T}\left[I({\hat{\theta }}_{\Omega })\right]\left({\hat{\theta }}_{\omega }-\theta_{\omega }\right)\;$in Eq. (15):

$$\begin{array}{c} {\left({\hat{\theta }}_{\omega }-\theta_{\omega }\right)}^{T}\left[I\left({\hat{\theta }}_{\Omega }\right)\right]\left({\hat{\theta }}_{\omega }-\theta_{\omega }\right)={\left[\begin{array}{c} 0_{p\left(K+1\right)\times \;1}\\ {\hat{\theta }}_{0\omega }-\theta_{0\omega } \end{array}\right]}^{T}\left[\begin{array}{cc} \left[I_{11}\right] & \left[I_{12}\right]\\ \left[I_{21}\right] & \left[I_{22}\right] \end{array}\right]\left[\begin{array}{c} 0_{p\left(K+1\right)\times \;1}\\ {\hat{\theta }}_{0\omega }-\theta_{0\omega } \end{array}\right]\\ =\left[\begin{array}{cc} {\left({\hat{\theta }}_{0\omega }-\theta_{0\omega }\right)}^{T}\left[I_{21}\right] & {\left({\hat{\theta }}_{0\omega }-\theta_{0\omega }\right)}^{T}\left[I_{22}\right] \end{array}\right]\left[\begin{array}{c} 0\\ {\hat{\theta }}_{0\omega }-\theta_{0\omega } \end{array}\right]\;\\ =0+\left[{\left({\hat{\theta }}_{0\omega }-\theta_{0\omega }\right)}^{T}\left[I_{22}\right]\left({\hat{\theta }}_{0\omega }-\theta_{0\omega }\right)\right]\; \end{array}$$

Corollary 1.2.3. Substitution$\;{\hat{\theta }}_{0\omega }={\hat{\theta }}_{2}+{\left[I_{22}\right]}^{-1}\left[I_{21}\right]{\hat{\theta }}_{1}$:

$$\begin{array}{c} \begin{array}{c} =\left[{\left({\hat{\theta }}_{2}-\theta_{0\omega }+{\left[I_{22}\right]}^{-1}\left[I_{21}\right]{\hat{\theta }}_{1}\right)}^{T}\left[I_{22}\right]\left({\hat{\theta }}_{2}-\theta_{0\omega }+{\left[I_{22}\right]}^{-1}\left[I_{21}\right]{\hat{\theta }}_{1}\right)\right]\\ =\left[{\left({\left[I_{22}\right]}^{-1}\left[I_{21}\right]{\hat{\theta }}_{1}+{\hat{\theta }}_{2}-\theta_{0\omega }\right)}^{T}\left[I_{22}\right]\left({\left[I_{22}\right]}^{-1}\left[I_{21}\right]{\hat{\theta }}_{1}+{\hat{\theta }}_{2}-\theta_{0\omega }\right)\right]\\ =\left[\left({\left({\hat{\theta }}_{1}\right)}^{T}\left[I_{12}\right]{\left({\left[I_{22}\right]}^{-1}\right)}^{T}\left[I_{22}\right]+{\left({\hat{\theta }}_{2}-\theta_{0\omega }\right)}^{T}\left[I_{22}\right]\right)\left(\left({\left[I_{22}\right]}^{-1}\left[I_{21}\right]{\hat{\theta }}_{1}\right)+\left({\hat{\theta }}_{2}-\theta_{0\omega }\right)\right)\right] \end{array}\\ \begin{array}{c} =\left[{\left({\hat{\theta }}_{1}\right)}^{T}\left[I_{12}\right]{\left[I_{22}\right]}^{-1}\left[I_{21}\right]{\hat{\theta }}_{1}+{\left({\hat{\theta }}_{2}-\theta_{0\omega }\right)}^{T}\left[I_{21}\right]\left({\hat{\theta }}_{1}\right)+{\left({\hat{\theta }}_{1}\right)}^{T}\left[I_{21}\right]\left({\hat{\theta }}_{2}-\theta_{0\omega }\right)+{\left({\hat{\theta }}_{2}-\theta_{0\omega }\right)}^{T}\left[I_{22}\right]\left({\hat{\theta }}_{2}-\theta_{0\omega }\right)\right]\\ ={\left[\begin{array}{c} {\hat{\theta }}_{1}\\ {\hat{\theta }}_{2}-\theta_{0\omega } \end{array}\right]}^{T}\left[\begin{array}{cc} \left[I_{12}\right]{\left[I_{22}\right]}^{-1}\left[I_{21}\right] & \left[I_{12}\right]\\ \left[I_{21}\right] & \left[I_{22}\right] \end{array}\right]\left[\begin{array}{c} {\hat{\theta }}_{1}\\ {\hat{\theta }}_{2}-\theta_{0\omega } \end{array}\right]\; \end{array}\; \end{array}$$

then,

(16) $${\left({\hat{\theta }}_{\omega }-\theta_{\omega }\right)}^{T}\left[I\left({\hat{\theta }}_{\Omega }\right)\right]\left({\hat{\theta }}_{\omega }-\theta_{\omega }\right)={\left[\begin{array}{c} {\hat{\theta }}_{1}\\ {\hat{\theta }}_{2}-\theta_{0\omega } \end{array}\right]}^{T}\left[\begin{array}{cc} \left[I_{12}\right]{\left[I_{22}\right]}^{-1}\left[I_{21}\right] & \left[I_{12}\right]\\ \left[I_{21}\right] & \left[I_{22}\right] \end{array}\right]\left[\begin{array}{c} {\hat{\theta }}_{1}\\ {\hat{\theta }}_{2}-\theta_{0\omega } \end{array}\right]\;$$

Substitute the results of (15) and (16) in Lemma 1.2 into Eq. (14), thus obtained:(17)

Eq. (17) is simplified to

$$G^{2}\approx \;{{\hat{\theta }}_{1}}^{T}{\left[I^{11}\right]}^{-1}{\hat{\theta }}_{1}$$

where,

$${\hat{\theta }}_{1}\overset{d}{\to }N\left(0,{\left[I^{11}\right]}_{\left(p\left(K+1\right)\times p\left(K+1\right)\right)}\right)$$

and

$${\left[I^{11}\right]}^{-\frac{1}{2}}\;{\hat{\theta }}_{1}\overset{d}{\to }N\left(0,I_{\left(p\left(K+1\right)\times p\left(K+1\right)\right)}\right)$$

Theorem 2. Under the null hypothesis$\;H_{0}$, the distribution of the Likelihood Ratio Test (LRT) statistic asymptotically follows a chi-square distribution withp(K+1)degrees of freedom, wherepis the number of parameters per component andKis the number of components.

Proof:

$$G^{2}\approx \;{\left[{\left[I^{11}\right]}^{-\frac{1}{2}}\;{\hat{\theta }}_{1}\right]}^{T}\left[{\left[I^{11}\right]}^{-\frac{1}{2}}\;{\hat{\theta }}_{1}\right]$$

$$G^{2}\approx \;Z^{T}Z\overset{d}{\to }\chi_{p\left(K+1\right)}^{2}$$

where $Z={\left[I^{11}\right]}^{-\frac{1}{2}}\;{\hat{\theta }}_{1}\overset{d}{\to }N\left(0,I_{\left(p(K+1)\right)}\right)$

Thus it can be concluded$\;G^{2}\overset{d}{\to }\chi_{p(K+1)}^{2}$

Theorem 3. Based on the statistical test defined in Theorem 1 and its distribution described in Theorem 2, the critical region for rejecting the null hypothesis$\;H_{0}\;$at a significance levelain the simultaneous hypothesis test of the parameters of the FSNBLR model is given by

$$G^{2}>\chi_{\left(a,p\left(K+1\right)\right)}^{2}$$

where$\;G^{2}\;$is the test statistic and$\;\chi_{\left(a,p(K+1)\right)}^{2}$​ is the upperaquantile of the chi-square distribution withp(K+1)degrees of freedom.

Proof:

$$\begin{array}{c} \begin{array}{c} a=P\left(\Lambda <\Lambda_{0}\right)\\ =P\left(\ln\Lambda^{2}<\ln{\Lambda_{0}}^{2}\right) \end{array}\\ \begin{array}{c} =P\left(-2\ln\Lambda >-2\ln\Lambda_{0}\right)\\ =P\left(-2\ln\Lambda >C\right) \end{array} \end{array}$$

$$a=P\left(G^{2}>\chi_{\left(a,p\left(K+1\right)\right)}^{2}\right)$$

Reject$\;H_{0}\;$if:

$$G^{2}>\chi_{\left(a,p\left(K+1\right)\right)}^{2}\;\text{or}\;P\left(G^{2}>\chi_{\left(a,p\left(K+1\right)\right)}^{2}\right)<a$$

#### Partial test

The Partial (individual) tests are conducted to determine the significance of parameters individually. The hypothesis form, parameter space, and statistic test are given as follows.

Hypothesis:

For the main effect parameters$\;b_{j}$, wherej=1,2…p

$$H_{0}:b_{j}=0\;\text{vs}\;H_{1}:b_{j}\neq 0$$

For the main effect parameters$\;a_{kj}$, wherek=1,2…K

$$H_{0}:a_{kj}=0\;\text{vs}\;H_{1}:a_{kj}\neq 0$$

Suppose the population MLE parameters are partitioned as:

$${\hat{\theta }}_{\Omega }={\left[\begin{array}{cc} {\hat{\theta }}_{1} & {\hat{\theta }}_{2} \end{array}\right]}^{T}$$

Where:

$${\hat{\theta }}_{1}=\left[{\hat{b}}_{1}\right]$$

$${\hat{\theta }}_{2}={\left[\begin{array}{ccc} \begin{array}{ccc} {a_{0}}^{*} & b_{1} & \cdots \end{array} & \begin{array}{ccc} b_{p} & a_{11} & \cdots \end{array} & \begin{array}{ccc} a_{K1} & \cdots & a_{Kp} \end{array} \end{array}\right]}^{T}$$

Under the null hypothesis$\;H_{0}$, the MLE parameters are partitioned as:

$${\hat{\theta }}_{\omega }={\left[\begin{array}{cc} {\hat{\theta }}_{0} & {\hat{\theta }}_{0\omega } \end{array}\right]}^{T}$$

where:

$${\hat{\theta }}_{0}={\left[\begin{array}{ccc} 0 & 0 & \begin{array}{cc} \cdots & 0 \end{array} \end{array}\right]}^{T}=0_{p\left(K+1\right)\times \;1}$$

$${\hat{\theta }}_{0\omega }={\left[\begin{array}{ccc} \begin{array}{ccc} {a_{0}}^{**} & {b_{1}}^{*} & \cdots \end{array} & \begin{array}{ccc} {b_{p}}^{*} & {a_{11}}^{*} & \cdots \end{array} & \begin{array}{ccc} {a_{K1}}^{*} & \cdots & {a_{Kp}}^{*} \end{array} \end{array}\right]}^{T}$$

Theorem 4. The test statistic used to evaluate a partial hypothesis, known as the Wald test, is given by

$$Z=\frac{{\hat{b}}_{j}}{\sqrt{Var\left({\hat{b}}_{j}\right)}},$$

where$\;{\hat{b}}_{j}\;$is the estimated coefficient of thej-th parameter, and$\;Var({\hat{b}}_{j})\;$is its estimated variance.

Proof:

The Wald test statistic is given by

(18) $$\begin{array}{c} \begin{array}{c} \chi^{2}\approx {\left({\hat{\theta }}_{1}-\theta_{0}\right)}^{T}\left(\left[I_{11}\right]-\left[I_{12}\right]{\left[I_{22}\right]}^{-1}\left[I_{21}\right]\right)\left({\hat{\theta }}_{1}-\theta_{0}\right)\;\\ \approx {\left({\hat{\theta }}_{1}-\theta_{0}\right)}^{T}{\left[I^{11}\right]}^{-1}\left({\hat{\theta }}_{1}-\theta_{0}\right) \end{array}\\ \begin{array}{c} \approx {\left({\hat{b}}_{j}-\theta_{0}\right)}^{T}{\left[I^{11}\right]}^{-1}\left({\hat{b}}_{j}-\theta_{0}\right)\\ \chi^{2}\approx {\hat{b}}_{j}^{2}{\left[I^{11}\right]}^{-1} \end{array} \end{array}$$

Lemma 4.1. Based on normal asymptotic properties:

$$\sqrt{n}\left(\hat{\theta }-E\left(\hat{\theta }\right)\right)\overset{d}{\to }N\left(0,{\left[I\left(\hat{\theta }\right)\right]}^{-1}\right)$$

where, according to [34]:

$$I\left(\hat{\theta }\right)=-E\left(H\left(\hat{\theta }\right)\right)\;\text{dan}\;{\left[I\left(\hat{\theta }\right)\right]}^{-1}={\left[-E\left(H\left(\hat{\theta }\right)\right)\right]}^{-1}$$

Corollary 4.1.1 The element$\;I^{11}=Var\left({\hat{b}}_{j}\right)\;$in Eq. (18) is the main diagonal element of the matrix$\;-{\left[H(\hat{\theta })\right]}^{-1}$

(19) $$\begin{array}{c} \chi^{2}\approx {\hat{b}}_{j}^{2}{\left[I^{11}\right]}^{-1}\\ \approx {\hat{b}}_{j}^{2}{\left[Var\left({\hat{b}}_{j}\right)\right]}^{-1}\\ \approx \frac{{\hat{b}}_{j}^{2}}{Var\left({\hat{b}}_{j}\right)}\overset{d}{\to }\chi_{\left(1\right)}^{2}\; \end{array}$$

Form of Eq. (19) is equivalent to a standard normal test:

$$Z=\frac{{\hat{b}}_{j}}{\sqrt{Var\left({\hat{b}}_{j}\right)}}\overset{d}{\to }N\left(0,1\right)$$

Corollary 4.1.2. Furthermore, with the same procedure, it can be done for hypothesis testing

$$H_{0}:a_{kj}=0\;,\;k=1,2\ldots K$$

$$H_{1}:a_{kj}\neq 0$$

Using the following test statistics:

$$Z=\frac{{\hat{a}}_{kj}}{\sqrt{Var\left({\hat{a}}_{kj}\right)}}$$

with$\;Var({\hat{a}}_{kj})\;$is the main diagonal element corresponding to$\;{\hat{a}}_{kj}\;$of the matrix$\;-{\left[H(\hat{\theta })\right]}^{-1}$

Theorem 5. Based on the statistical test and its distribution described in Theorem 4, the critical region for rejecting the null hypothesis$\;H_{0}\;$at a significance levelain the partial hypothesis test of the parameters of the FSNBLR model is given by

$$\left|Z\right|>Z_{a\;/\;2}$$

whereZis the Wald test statistic, and$\;Z_{a\;/\;2}\;$is the uppera/2quantile of the standard normal distribution.

Proof:

Since the statistical test given in Theorem 4, critical area for rejection of$\;H_{0}\;$is obtained as follows:

$$\begin{array}{c} a=P\left(\chi^{2}>C\right)\;\\ =P\left(Z\langle -\sqrt{C}\;atau\;Z\rangle \sqrt{C}\right)\; \end{array}$$

$$=P\left(Z\langle -Z_{a\;/\;2}\;atau\;Z\rangle Z_{a\;/\;2}\right)\;\text{with}\;Z_{a\;/\;2}=\sqrt{C}$$

$$a=P\left(\left|Z\right|>Z_{a\;/\;2}\right)$$

Reject$\;H_{0}\;$if:

$$\left|Z\right|>Z_{a\;/\;2}\;\text{or}\;P\left(\left|Z\right|>Z_{a\;/\;2}\right)<a$$

### Data collection

In applying the FSNBLR method, this study employs secondary data concerning the status of underdeveloped regions in Eastern Indonesia for the year 2021. The dataset includes one binary response variable, indicating whether a region is classified as underdeveloped or not. Then, six continuous predictor variables, obtained from various official sources such as *Kementerian Desa, Pembangunan Daerah Tertinggal, dan Transmigrasi; Kementerian Dalam Negeri; Kementerian Pendidikan, Kebudayaan, Riset, dan Teknologi; Kementerian Keuangan*; and *Badan Pusat Statistik*.

### Research variabel

The data consists of one response variable (y) and six predictor variables (x). The variables are detailed in Table 1.

**Table 1:** Variable description.

Variable	Notation	Description	Unit	Scale
Response	y	Status of Underdeveloped Regions	0 = Developed 1 = Underdeveloped	Nominal
Predictor	$x_{1}$	Percentage of Households Utilizing Clean Water	Percent	Ratio
	$x_{2}$	Percentage of Villages with the Widest Primary Roads Surfaced by Asphalt or Concrete	Percent	Ratio
	$x_{3}$	Percentage of Villages without Disaster	Percent	Ratio
	$x_{4}$	GDRB per Capita	Thousand rupiahs	Ratio
	$x_{5}$	Percentage Senior High School Enrollment Rate	Percent	Ratio
	$x_{6}$	PAD per Capita	Hundred thousand rupiahs	Ratio

**Fig. 1:**
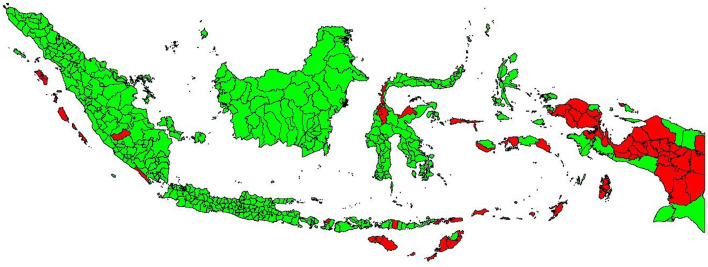
Distribution of underdeveloped regions in Indonesia in 2021.

Fig. 1 depicts a map of the spatial distribution of underdeveloped regions.

Based on Fig. 1, there are a total of 62 underdeveloped regencies across Indonesia. Of these, 55 are located in Eastern Indonesia, as detailed in Table 2.

**Table 2:** List of underdeveloped areas in Eastern Indonesia 2021 based on presidential regulation No 63/2020.

Region	Province	District	Total
Papua	Papua Barat	Manokwari Selatan, Maybrat, Pegunungan Arfak, Sorong, Sorong Selatan, Tambrauw, Teluk Bintuni, and Teluk Wondama.	8 Districts
	Papua	Asmat, Boven Digoel, Deiyai, Dogiyai, Intan Jaya, Jayawijaya, Keerom, Lanny Jaya, Mappi, Memberamo Raya, Memberamo Tengah, Nabire, Nduga, Paniai, Pegunungan Bintang, Puncak, Puncak Jaya, Supiori, Tolikara, Waropen, Yahukimo, and Yalimo.	22 Districts
Maluku	Maluku	Maluku Tenggara Barat, Kepulauan Aru, Seram Bagian Barat, Seram Bagian Timur, Maluku Barat Daya, Buru Selatan	6 Districts
	Maluku Utara	Kepulauan Sula, Pulau Taliabu.	2 Districts
Nusa Tenggara	NTB	Lombok Utara	1 District
	NTT	Alor, Belu, Kupang, Lembata, Malaka, Manggarai Timur, Rote Ndao, Sabu Raijua, Sumba Barat, Sumba Barat Daya, Sumba Tengah, Sumba Timur, and Timor Tengah Selatan.	13 Districts
Sulawesi	Sulawesi Tengah	Donggala, Tojo Una-Una, Sigi	3 Districts
Total	11 Provinces	55 Districts	

## Method validation

### Descriptive analytics

Descriptive analysis is conducted to understand the characteristics of each predictor variables, as presented in Table 3.

**Table 3:** Descriptive statistics of the predictor variables.

Variable	Mean	Variance	Min	Max
$x_{1}$	81.28	373.37	0.00	100.00
$x_{2}$	69.14	821.01	0.00	100.00
$x_{3}$	55.90	681.78	0.00	100.00
$x_{4}$	55.20	3937.56	6.18	589.11
$x_{5}$	63.89	246.68	8.25	94.92
$x_{6}$	46.93	1191.44	4.81	281.79

Table 3 summarized the descriptive statistics for the six predictor variable used in the study, which represent characteristics of 232 regencies/cities in Eastern Indonesia. These variables are related to the classification of underdeveloped regions. All independent variables are correlated with the dependent variable [28], contain no missing values [29], and show no indication of multicollinearity [30]. The multicollinearity test is shown in the Table 4 below.

**Table 4:** Multicolinearity test of the predictor variables.

Variable	VIF
$x_{1}$	1.27
$x_{2}$	1.37
$x_{3}$	1.13
$x_{4}$	1.59
$x_{5}$	1.34
$x_{6}$	1.58

Based on Table 4, each predictor variable has a VIF value of <10, so it can be concluded that there is no multicollinearity between the predictor variables in the model. Each predictor variable is defined in accordance with Ministerial Regulation No 11/2020, which described as follows:

1. Percentage of Households Utilizing Clean Water, is the proportion of households in a district/city that utilize clean water, calculated as the number of such households relative to the total, expressed as a percentage.
2. Percentage of Villages with the Widest Primary Roads Surfaced by Asphalt or Concrete, is calculated by dividing the number of villages with asphalt or concrete as the main road surface type by the total number of villages in the district.
3. Proportion of Villages Free from Natural Disasters*,* is the ratio of villages that have not experienced natural disasters (e.g., floods, earthquakes, droughts) within the past three years, expressed in percentage terms relative to the total number of villages.
4. GRDP per Capita, refers to the Gross Regional Domestic Product measured on a per-person basis within a district.
5. Percentage Senior High School Enrollment Rate, is the proportion of individuals aged 16–18 currently enrolled in senior high school compared to the total population in that age group, presented as a percentage.
6. PAD per Capita, is the district’s own-source revenue divided by its total population, indicating the fiscal capacity per person.

**Fig. 2:**
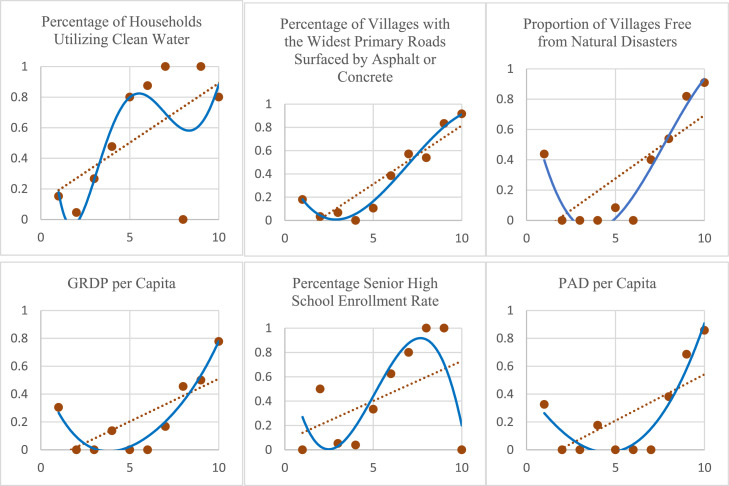
Scatterplots comparing multiple data groups against proportion of underdeveloped regions per group.

To explore the potential nonlinear relationships between each predictor and underdeveloped status, we constructed scatterplots of grouped data against the proportion of underdeveloped regions. These are presented in Fig. 2.

From Fig. 2, it is evident that the probability of being underdeveloped tends to increase with each predictor variable, following a repetitive and ascending pattern. This supports the suitability of modeling the data using a FSNBLR approach. Accordingly, this study proceed by implementing both the Binary Logistic Regression (BLR) and FSNBLR models. The parameter estimation and their statistical significance are discussed in the following section.

### BLR model

The BLR model is specified as follows.

$$\pi \left(x_{i}\right)=\;\frac{\exp\left(\beta_{0}+\beta_{1}x_{1}+\beta_{2}x_{2}+\ldots +\beta_{p}x_{p}\right)}{1+\exp\left(\beta_{0}+\beta_{1}x_{1}+\beta_{2}x_{2}+\ldots +\beta_{p}x_{p}\right)}\;;\;i=1,2...n$$

where,$\;\beta_{0}\;$is the intercept, and$\;\beta_{j}\;,\;j=1,2\ldots \;p\;$are the slope coefficients corresponding to the predictor variables.

#### Parameter estimation in BLR model

Based on the estimation output, the fitted BLR model for underdeveloped regions data is given by:

$$\hat{\pi }\left(x_{i}\right)=\;\frac{\exp\left(3.6276-0.0084x_{1i}-0.0438x_{2i}+0.0128x_{3i}-0.0121x_{4i}-0.0072x_{5i}\;-0.0301x_{6i}\right)}{1+\exp\left(3.6276-0.0084x_{1i}-0.0438x_{2i}+0.0128x_{3i}-0.0121x_{4i}-0.0072x_{5i}\;-0.0301x_{6i}\right)}$$

#### Significant parameter in BLR model

The significance test for each parameter in the model is shown in Table 5.

**Table 5:** Significant parameter in BLR model.

Parameters	Estimations	Std. Error	z value	Pr(>|z|)	Decision
$\beta_{0}$	3.6276	1.5368	2.36	0.0183	reject$\;H_{0}$
$\beta_{1}$	−0.0085	0.0125	−0.676	0.4993	fail to reject$\;H_{0}$
$\beta_{2}$	−0.0438	0.0096	−4.555	0.000005	reject$\;H_{0}$
$\beta_{3}$	0.0128	0.0087	1.456	0.1455	fail to reject$\;H_{0}$
$\beta_{4}$	−0.0121	0.0105	−1.148	0.2509	fail to reject$\;H_{0}$
$\beta_{5}$	−0.0072	0.0180	−0.4	0.6894	fail to reject$\;H_{0}$
$\beta_{6}$	−0.0301	0.0140	−2.146	0.0319	reject$\;H_{0}$

The results in Table 5 indicated that only$\;x_{2}\;$(percentage of villages with asphalt/concrete roads) and$\;x_{6}\;$(PAD per capita) are statistically significant at the 5 % significance level. Therefore, these two variables significantly influence the probability of a region being classified as underdeveloped.

The refined model using only statistically significant parameters is:

$$\hat{\pi }\left(x_{i}\right)=\;\frac{\exp\left(3.4452-0.0490x_{2i}\;-0.0414x_{6i}\right)}{1+\exp\left(3.4452-0.0490x_{2i}\;-0.0414x_{6i}\right)}$$

Detailed parameter estimation for the best BLR model using only statistically significant parameters provided in Table 6.

**Table 6:** Parameter estimation of the best BLR model.

Parameters	Estimations	Std. Error	z value	Pr(>|z|)	Decision
$\beta_{0}$	3.4452	0.6515	5.288	0.00000012	reject$\;H_{0}$
$\beta_{2}$	−0.0490	0.0079	−6.177	0.000000000653	reject$\;H_{0}$
$\beta_{6}$	−0.0414	0.0109	−3.800	0.000145	reject$\;H_{0}$

Based on Table 6, this model suggests that higher percentages of asphalt/concrete road coverage and greater PAD per capita are associated with a lower probability of a region being underdeveloped.

### FSNBLR model

#### Selection of optimal parameters

The oscillation parameters in the FSNBLR model were determined through the minimization of the Akaike Information Criterion (AIC). In order to avoid overfitting and to ensure the interpretability of the model, the number of oscillation parameters were limited. Using an R algorithm, the AIC values for various combinations of oscillation parameters were calculated and are summarized in Table 7.

**Table 7:** Minimum AIC values for each number of oscillation parameter.

Maximum Parameter Oscillation	Combination (K)						AIC (K)
	$x_{1}$	$x_{2}$	$x_{3}$	$x_{4}$	$x_{5}$	$x_{6}$	
K=1	1	1	1	1	1	1	169.3606
K=2	1	2	1	1	1	1	169.1771
K=3	3	2	1	1	1	1	168.4055

Based on Table 7, the model with oscillation parameters$\;(x_{1}=3,\;x_{2}=2,x_{3}=1,x_{4}=1,x_{5}=1,x_{6}=1)\;$yields the smallest AIC and is therefore selected as the optimal FSNBLR model.

#### Parameter estimation in FSNBLR model

The FSNBLR model (25) can be expressed as:

$$\hat{\pi }\left(x_{i}\right)=\;\frac{\exp\left(4.03-0.0005x_{1i}+0.09\cos x_{1i}+0.40\cos2x_{1i}-0.55\cos3x_{1i}+\ldots -0.03x_{6i}+0.08\cos x_{6i}\right)}{1+\exp\left(4.03-0.0005x_{1i}+0.09\cos x_{1i}+0.40\cos2x_{1i}-0.55\cos3x_{1i}+\ldots -0.03x_{6i}+0.08\cos x_{6i}\right)}$$

Detailed parameter estimation are provided in Table 8.

**Table 8:** Parameter estimation of the FSNBLR model.

Parameters	Estimations	Parameters	Estimations
$a_{0}$	4.0393	$b_{3}$	0.0115
$b_{1}$	0.0005	$a_{1,3}$	−0.4500
$a_{1,1}$	0.0946	$b_{4}$	−0.0092
$a_{2,1}$	0.4071	$a_{1,4}$	−0.3481
$a_{3,1}$	−0.5541	$b_{5}$	−0.0117
$b_{2}$	−0.0582	$a_{1,5}$	0.3969
$a_{1,2}$	−0.5454	$b_{6}$	−0.0338
$a_{2,2}$	−0.5136	$a_{1,6}$	0.0831

#### Significant parameter in FSNBLR model

Significance tests for each parameter are summarized in Table 9.

**Table 9:** Significant test of parameters in the FSNBLR model.

Parameters	Estimations	Std. Error	z value	Pr(>|z|)	Decision
$a_{0}$	4.0393	1.6536	2.443	0.0146	reject$\;H_{0}$
$b_{1}$	0.0005	0.0129	0.040	0.9685	fail to reject$\;H_{0}$
$a_{1,1}$	0.0946	0.3222	0.294	0.7689	fail to reject$\;H_{0}$
$a_{2,1}$	0.4071	0.3377	1.205	0.2280	fail to reject$\;H_{0}$
$a_{3,1}$	−0.5541	0.3123	−1.774	0.0760	fail to reject$\;H_{0}$
$b_{2}$	−0.0582	0.0121	−4.787	0.000001	reject$\;H_{0}$
$a_{1,2}$	−0.5454	0.3469	−1.572	0.1160	fail to reject$\;H_{0}$
$a_{2,2}$	−0.5136	0.3463	−1.483	0.1381	fail to reject$\;H_{0}$
$b_{3}$	0.0115	0.0095	1.212	0.2254	fail to reject$\;H_{0}$
$a_{1,3}$	−0.4500	0.3339	−1.348	0.1778	fail to reject$\;H_{0}$
$b_{4}$	−0.0092	0.0085	−1.085	0.2779	fail to reject$\;H_{0}$
$a_{1,4}$	−0.3481	0.3401	−1.023	0.3061	fail to reject$\;H_{0}$
$b_{5}$	−0.0117	0.0207	−0.567	0.5706	fail to reject$\;H_{0}$
$a_{1,5}$	0.3969	0.3431	1.157	0.2472	fail to reject$\;H_{0}$
$b_{6}$	−0.0338	0.0143	−2.360	0.0183	reject$\;H_{0}$
$a_{1,6}$	0.0831	0.3216	0.259	0.7959	fail to reject$\;H_{0}$

Based on Table 9, the results indicate that only$\;x_{2}\;$(percentage of villages with asphalt/concrete roads) and$\;x_{6}\;$(PAD per capita) are statistically significant.

The best FSNBLR model with significant parameters and oscillation parameters$\;(x_{2}=2,\;x_{6}=3)\;$is:

$$\hat{\pi }\left(x_{i}\right)=\;\frac{\exp\left(3.76-0.05x_{2i}-0.47\cos x_{2i}-0.51\cos2x_{2i}-0.04x_{6i}+0.23\cos x_{6i}-0.01\cos2x_{6i}-0.66\cos3x_{6i}\right)}{1+\exp\left(3.76-0.05x_{2i}-0.47\cos x_{2i}-0.51\cos2x_{2i}-0.04x_{6i}+0.23\cos x_{6i}-0.01\cos2x_{6i}-0.66\cos3x_{6i}\right)}$$

Detailed parameter estimation for the best FSNBLR model using only statistically significant parameters provided in Table 10.

**Table 10:** Parameter estimation of the best BLR model.

Parameters	Estimations	Std. Error	z value	Pr(>|z|)	Decision
$a_{0}$	3.7695	0.7187	5.245	0.00000015	reject$\;H_{0}$
$b_{2}$	−0.0539	0.0089	−6.034	0.0000000016	reject$\;H_{0}$
$a_{1,2}$	−0.4783	0.3184	−1.502	0.1330	fail to reject$\;H_{0}$
$a_{2,2}$	−0.5151	0.3161	−1.629	0.1032	fail to reject$\;H_{0}$
$b_{6}$	−0.0440	0.0113	−3.882	0.00010	reject$\;H_{0}$
$a_{1,6}$	0.2373	0.3020	0.786	0.4319	fail to reject$\;H_{0}$
$a_{2,6}$	−0.0105	0.2882	−0.036	0.9709	fail to reject$\;H_{0}$
$a_{3,6}$	−0.6604	0.3356	−1.968	0.0490	Reject H_0_

Based on Table 10, this implies that the status of underdeveloped regions is significantly influenced by the percentage of villages with asphalt/concrete roads and PAD per capita, including their oscillatory effects captured by the Fourier series components.

### Comparison of BLR and FSNBLR

#### Model selection for classification based on deviance value

The preferred regression model is the one with the lowest deviance value. The results obtained from the deviance statistical test are presented in Table 11.

**Table 11:** Deviance value comparison between models.

Methods	Deviance Values
BLR	157.6297
FSNBLR	147.4715

Based on Table 11, the deviance value for the FSNBLR (147.4715) was smaller than that for the BLR (157.6297). Therefore, the FSNBLR model is the best model for data on the status of underdeveloped regions because has the smallest deviance value.

#### Model selection for classification based on AUC and Press’s Q value

The chosen FSNBLR model exhibited the highest AUC or the lowest Press's Q value. The results of the classification test are presented in Table 12.

**Table 12:** AUC and Press’s Q value comparison between models.

Methods	Accuracy	Sensitivity	Specificity	AUC	Press’s Q	Chi Square
BLR	82.32 %	92.65 %	49.09 %	70.87 %	96.9827	96.4927
FSNBLR	84.05 %	93.22 %	54.54 %	73.88 %	107.6034	96.4927

Table 12 indicates that the FSNBLR model achieved a higher AUC value (73.88 %) compared to the BLR model (70.87 %). Furthermore, the greater Press’s Q value for FSNBLR (107.6034) suggests a stronger classification capability and a higher likelihood of rejecting the null hypothesis.

## Summary

Based on the hypothesis testing and model comparisons that had been conducted, the BLR model identified variables$\;x_{2}\;$(Percentage of Villages with Asphalt/Concrete Road Surface) and$\;x_{6}\;$(PAD Per-Capita) as significant parameters. This is also the same as the FSNBLR model, which found$\;x_{2}\;$and$\;x_{6}\;$to be significant. The FSNBLR model with significant parameters for categorical data is followed:

$$\hat{\pi }\left(x_{i}\right)=\;\frac{\exp\left(3.76-0.05x_{2i}-0.47\cos x_{2i}-0.51\cos2x_{2i}-0.04x_{6i}+0.23\cos x_{6i}-0.01\cos2x_{6i}-0.66\cos3x_{6i}\right)}{1+\exp\left(3.76-0.05x_{2i}-0.47\cos x_{2i}-0.51\cos2x_{2i}-0.04x_{6i}+0.23\cos x_{6i}-0.01\cos2x_{6i}-0.66\cos3x_{6i}\right)}$$

The FSNBLR model provided better fit and classification performance than the BLR model, as shown by higher AUC, accuracy, sensitivity, and specificity values. Therefore, it was concluded that the FSNBLR model was more suitable for predicting the status of underdeveloped regions in Eastern Indonesia in 2021. The results shown that the significant parameters are the Percentage of Villages with Asphalt/Concrete Road Surface and PAD Per-Capita. These two variables should also be considered as key elements in planning development policies to reduce regional underdevelopment.

A higher percentage of villages with asphalt or concrete road surfaces indicates better transportation infrastructure, which plays a critical role in supporting economic activities. Good road conditions facilitate the smooth distribution of goods and services, ease access to markets, reduce travel time and costs, and improve connectivity between regions. This, in turn, stimulates economic growth and development in other sectors such as agriculture, trade, and education.

Meanwhile, a higher PAD per capita reflects the fiscal capacity of a region. With greater locally-generated revenue, local governments have more flexibility and resources to fund strategic programs—such as infrastructure development, education, health services, and economic empowerment initiatives. This financial capacity is essential in supporting comprehensive and sustainable efforts to uplift underdeveloped areas. Therefore, strengthening road infrastructure and increasing PAD should be prioritized as part of integrated regional development strategies.

These findings have direct socioeconomic and policy implications. The statistical significance of infrastructure and fiscal capacity variables highlights the need for development strategies that prioritize equitable budget allocation toward road improvement and local revenue enhancement. In practical terms, this means that national and regional governments should integrate infrastructure investment and fiscal capacity-building programs into a coordinated regional development agenda—ensuring that remote districts in Eastern Indonesia receive both physical connectivity and financial autonomy to sustain local growth. By translating the FSNBLR results into actionable policy priorities, the study bridges quantitative modeling with real-world decision-making in regional development planning.

Nevertheless, the study acknowledges several limitations, including potential data heterogeneity, temporal instability, and challenges in parameter interpretability. Future research is encouraged to extend the FSNBLR framework to panel and spatial data structures, or to integrate it with AI-based prediction techniques, to enhance robustness and capture the evolving dynamics of regional inequality. Furthermore, extending the FSNBLR framework to account for spatial dependencies or uncertainty quantification via Bayesian inference represents another promising direction to improve model reliability and applicability in complex socioeconomic data settings. Overall, the FSNBLR framework provides a strong methodological foundation for future interdisciplinary research that links quantitative modeling, spatial analysis, and policy design—supporting more adaptive and evidence-based regional development planning in Indonesia and comparable developing economies.

## Limitations

1. The method used to select the optimal oscillation parameters was the smallest AIC.
2. The number of oscillation parameters (k) used in the Fourier series in this study is k = 1, 2, and 3
3. The estimation method used in the Fourier series nonparametric regression model of categorical data is Maximum Likelihood Estimation (MLE).
4. The cutoff threshold for classifying the results 0 and 1 from the probability prediction used is 0.5.
5. The hypothesis testing method used is the Maximum Likelihood Ratio Test (MLRT).
6. The confidence interval method used is Pivotal Quantity.
7. The best model-comparison method uses the classification test.
8. The data used are secondary data from Eastern Indonesia (West Nusa Tenggara, East Nusa Tenggara, West Kalimantan, Central Kalimantan, South Kalimantan, East Kalimantan, North Kalimantan, North Sulawesi, Central Sulawesi, South Sulawesi, Southeast Sulawesi, Gorontalo, West Sulawesi, Maluku, North Maluku, West Papua, and Papua).
9. Ignoring dependencies between observations, so that observation groups can be obtained from the smallest to largest sequence of values.
10. Continuous variables do not exhibit multicollinearity.

## Ethics statements

The data we use in this research are secondary data that we collected from publications of each province in Eastern Indonesia. The data is available on request.

## CRediT authorship contribution statement

**Muhammad Zulfadhli:** Conceptualization, Methodology, Software, Writing – original draft, Visualization. **I Nyoman Budiantara:** Conceptualization, Methodology, Writing – review & editing, Validation, Supervision. **Vita Ratnasari:** Conceptualization, Methodology, Writing – review & editing, Validation, Supervision. **Afiqah Saffa Suriaslan:** Conceptualization, Methodology, Writing – review & editing, Validation, Supervision. **Risdiana Chandra Dhewy:** Conceptualization, Methodology, Writing – review & editing, Validation, Supervision.

## Declaration of competing interest

The authors declare that they have no known competing financial interests or personal relationships that could have appeared to influence the work reported in this paper.

## Data Availability

The authors do not have permission to share data.
